# Reactivation of Multiple Viruses in Patients with Sepsis

**DOI:** 10.1371/journal.pone.0098819

**Published:** 2014-06-11

**Authors:** Andrew H. Walton, Jared T. Muenzer, David Rasche, Jonathan S. Boomer, Bryan Sato, Bernard H. Brownstein, Alexandre Pachot, Terrence L. Brooks, Elena Deych, William D. Shannon, Jonathan M. Green, Gregory A. Storch, Richard S. Hotchkiss

**Affiliations:** 1 Department of Anesthesiology, Washington University School of Medicine, St. Louis, Missouri, United States of America; 2 Department of Pediatrics, Washington University School of Medicine, St. Louis, Missouri, United States of America; 3 Department of Medicine, Washington University School of Medicine, St. Louis, Missouri, United States of America; 4 Department of Surgery, Washington University School of Medicine, St. Louis, Missouri, United States of America; 5 Medical Diagnostic Discovery Department, BioMérieux Inc., Marcy 1'Etoile, France; University of Florida College of Medicine, United States of America

## Abstract

A current controversy is whether patients with sepsis progress to an immunosuppressed state. We hypothesized that reactivation of latent viruses occurred with prolonged sepsis thereby providing evidence of clinically-relevant immunosuppression and potentially providing a means to serially-monitor patients' immune status. Secondly, if viral loads are markedly elevated, they may contribute to morbidity and mortality. This study determined if reactivation of herpesviruses, polyomaviruses, and the anellovirus TTV occurred in sepsis and correlated with severity. Serial whole blood and plasma samples from 560 critically-ill septic, 161 critically-ill non-septic, and 164 healthy age-matched patients were analyzed by quantitative-polymerase-chain-reaction for cytomegalovirus (CMV), Epstein-Barr (EBV), herpes-simplex (HSV), human herpes virus-6 (HHV-6), and TTV. Polyomaviruses BK and JC were quantitated in urine. Detectable virus was analyzed with respect to secondary fungal and opportunistic bacterial infections, ICU duration, severity of illness, and survival. Patients with protracted sepsis had markedly increased frequency of detectable virus. Cumulative viral DNA detection rates in blood were: CMV (24.2%), EBV (53.2%), HSV (14.1%), HHV-6 (10.4%), and TTV (77.5%). 42.7% of septic patients had presence of two or more viruses. The 50% detection rate for herpesviruses was 5–8 days after sepsis onset. A small subgroup of septic patients had markedly elevated viral loads (>10^4^–10^6^ DNA copies/ml blood) for CMV, EBV, and HSV. Excluding TTV, DNAemia was uncommon in critically-ill non-septic patients and in age-matched healthy controls. Compared to septic patients without DNAemia, septic patients with viremia had increased fungal and opportunistic bacterial infections. Patients with detectable CMV in plasma had higher 90-day mortality compared to CMV-negative patients; *p*<0.05. Reactivation of latent viruses is common with prolonged sepsis, with frequencies similar to those occurring in transplant patients on immunosuppressive therapy and consistent with development of an immunosuppressive state. Whether reactivated latent viruses contribute to morbidity and mortality in sepsis remains unknown.

## Introduction

Sepsis is the host's non-resolving inflammatory response to infection that leads to organ dysfunction [1], [2]. A current controversial hypothesis postulates that if sepsis pursues a protracted course, it progresses from an initial primarily hyper-inflammatory phase to a predominantly immunosuppressive state [3]–[7]. Experimental therapeutic approaches in sepsis have almost exclusively focused on blocking early inflammation or host-pathogen interaction and failed [8]–[10]. Recently, immuno-adjuvant therapies that boost host immunity, e.g., GM-CSF and interferon-γ, have been successful in small clinical trials thereby supporting the concept that reversing immunosuppression in sepsis is a plausible strategy to improve outcome [11], [12]. However, several issues have limited this approach including lack of consensus that immunosuppression is a clinically important phenomenon [5], [6], [13]. Also, difficulty in identifying patients with impaired immunity as well as determining optimal timing for administration pose significant challenges to pursuing this approach [14]. While immuno-adjuvant therapies might improve sepsis survival if administered during the later immunosuppressive phase, these agents might worsen outcome if given during the early hyper-inflammatory phase [4], [14]. Thus, a means to distinguish these two contrasting phases of sepsis is needed not only to verify the hypothesis that sepsis progresses to an immunosuppressive state but also to guide use of potential agents which boost immunity.

Latent viruses such as cytomegalovirus are normally held in abeyance by cellular and immune surveillance mechanisms which if impaired, for example by immunosuppressive medications, often result in viral reactivation, replication, and virally-mediated tissue injury [15]–[20]. Sepsis impairs innate and adaptive immunity by multiple mechanisms including apoptosis-induced depletion of immune effector cells and induction of T-cell exhaustion thereby possibly predisposing to viral reactivation and dissemination [21]–[23].

Although viral reactivation has been documented in sepsis, studies have generally been limited in scope, focusing on CMV viremia or HSV-1 pneumonitis [15], [18], [20], [24]–[28]. No comprehensive study of the herpes or polyomavirus family has been conducted in sepsis. Demonstration that widespread reactivation of latent herpes and polyomavirus occurs in sepsis has several important implications. First, it would provide strong evidence that sepsis results in functional immunosuppression and may provide a means to track patient immunocompetence during the disorder. Secondly, depending upon the level of viremia, reactivated viruses may contribute to morbidity and mortality in the disorder. We also investigated TTV, an anellovirus previously shown to be present in up to 40–50% of healthy adults [29]–[33]. Recent studies in patients with liver and stem cell transplantation, patients with HIV, and patients with chronic renal failure indicate that the magnitude of TTV viremia reflects patient immunocompetence and that TTV viral load is useful as a surrogate marker of the robustness of immunity [30]–[33].

## Methods: (in addition, see Supporting Information)

### Inclusion criteria

#### Septic patients

Non-immunocompromised patients treated in surgical/medical ICUs (2009–2013) were identified prospectively. Sepsis was defined as a microbiologically-proven, clinically-proven, or suspected infection and presence of systemic inflammatory response syndrome [10]. Patients were followed through hospital discharge or 90 days after sepsis onset. Mortality status at 90 days was available for >95% of study subjects.

#### Critically-ill non-septic patients (CINS) and healthy-control patients

Non-septic, non-immunocompromised patients being treated in surgical/medical ICUs were one comparison group. A second group consisted of age-matched, ambulatory, pre-operative elective-surgery patients (American Society of Anesthesiology [ASA] class 1–3).

### Exclusion criteria

Patient exclusion criteria included: HIV-1, organ transplantation, high-dose corticosteroids (≥300 mgs/day hydrocortisone) or other immunosuppressive medications, viral hepatitis, and autoimmune diseases.

### Blood and Urine Collection

Analyses were performed on residual blood remaining after clinical hematologic testing was performed (Septic and CINS), or blood obtained from ambulatory volunteers prior to elective surgery (Healthy Control). Blood was retrieved daily starting within 24–72 hrs. of ICU admission. Whole blood and plasma were stored at −80°C. For detection of BK and JC, urine was typically obtained twice/week. Quantitative-PCR (qPCR) was performed 2–3 times/week.

### CMV Serologic testing

IgG antibodies to CMV were quantitated by ELISA to identify individuals with prior CMV exposure.

### Sample preparation and viral DNA detection

DNA was extracted using the NucliSens-EasyMag-extractor (BioMérieux) and assayed for viruses by qPCR using protocols from the Clinical Virology Laboratory at St Louis Children's Hospital (except HHV-6 and TTV) and as previously described [34]–[39]. Briefly 5 uL of sample was used per reaction, and assays were performed on either an ABI 7500 Fast system (Applied Biosystems), or a LightCycler II (Roche). (See Table S1. for details regarding lower limit of quantitation for each virus and interassay coefficient-of-variation).

### Statistical analysis

Data were analyzed using SAS-Statistical Software. Kaplan-Meier analyses were used for mortality, ICU length-of-stay, and secondary infection rates. Chi-square and *t*-tests were used for categorical and continuous variables.

### Human Studies Human Studies

The study was approved by the Washington University Human Research Protection Office. Patient consent was obtained for venipuncture and chart review from pre-operative elective surgery patients. Oral consent was documented by having the patient sign the study consent form which was then placed in the patient chart with an additional copy kept with the research nurse coordinator. For septic and critically-ill non-septic patients, a waiver of consent was granted for obtaining excess clinical “waste” laboratory blood (that was slated to be discarded) and for review of their relevant hospital records because these procedures were considered to represent minimum risk to the patients.

## Results

### Demographic data

560 septic, 160 CINS, and 165 healthy control patients were included (Table 1). The 560 septic patients included 31 patients originally classified as CINS who developed sepsis during their ICU admission and were transferred into the septic category. Median duration of ICU stay was 11 days (range 2–127) and 2 days (range 1–12) for septic and CINS patients respectively. The number of blood samples for septic and CINS patients ranged from 1–27 (mean 3.1) and 1–2 (mean 1.1) respectively. A single blood sample was obtained prior to surgery for the healthy control patients.

**Table 1:** Patient Characteristics.

		Septic	Critically-Ill Non-Septic	Healthy Controls
*# Patients*		560	160	165
Age	Median	63	63	64
	range[IQR]	52–74	53–76	60–72
Gender (%)	Male	305 (55)	81 (51)	81 (49)
	Female	255 (45)	79 (49)	84 (51)
Apache II *	median	18	5	
	range[IQR]	15–22	4–7	
SOFA**	median	7	2	
	range[IQR]	5–10	1–3	
Length of ICU Stay	median	11	2	
	range[IQR]	6–19	2–3	
Mortality (%)	survived	416 (74)	151 (94)	
	expired	144 (26)	9 (6)	
Admission ICU Diagnosis	Trauma		59	
	Post-operation (major surgery)		37	
	Neurologic events		40	
	Cardiovascular events		7	
	Miscellaneous		17	
Site of Infection	Pneumonia	284		
	Peritonitis	181		
	Surgical site and wound infection	71		
	Intravascular catheter infection	14		
	Urinary tract infection	10		

*Apache II, “Acute Physiology and Chronic Health Evaluation II” at ICU admission.

**SOFA, “Sequential Organ Failure Assessment” at ICU admission.

### Cumulative detection rates and levels of herpes family viruses

#### CMV

**Figure 1:**
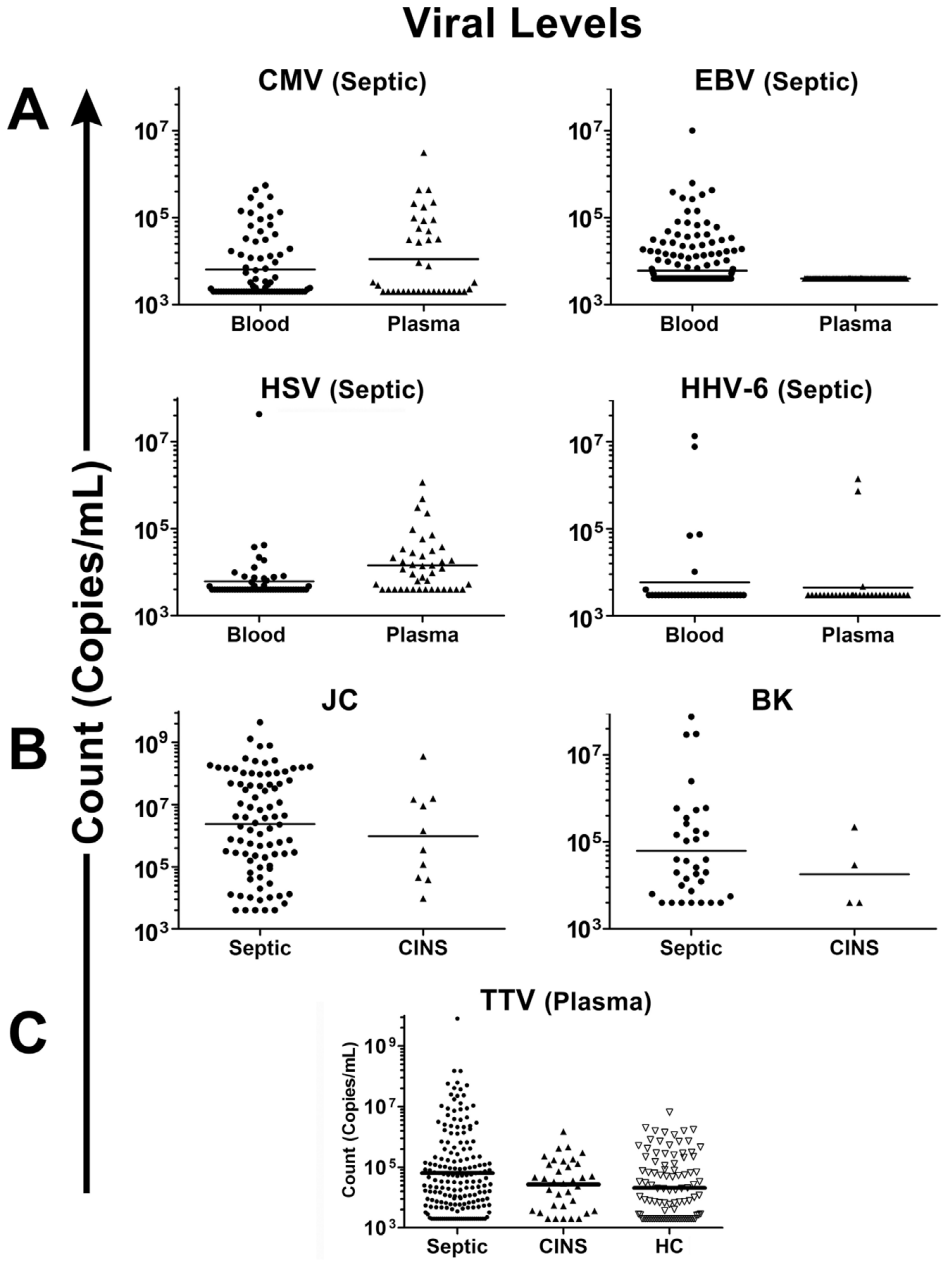
Viral levels in septic and control patients. The maximum viral load for each patient is displayed. (Figure 1A) Only data from septic patients are displayed for CMV, EBV, HSV, and HHV-6 because viral levels in control patients for these viruses were at or below the limit of quantitation. (*Figure 1B*) For JC and BK, data are from urine samples of septic and critically-ill non-septic (CINS) patients. (*Figure 1C*) The maximum viral load for TTV is displayed for septic, CINS, and healthy control pre-operative elective-surgery (HC) patients. The horizontal line in each graph represents the geometric mean for the virus level.

70.2% of patients (septic and controls) were CMV seropositive within 2–4 days of ICU admission, indicative of prior infection. With one exception, detection of CMV by PCR occurred only in patients who were CMV seropositive. 24.2% of septic CMV seropositive patients had CMV detected with geometric mean (geomean) levels of 6,409 copies/ml whole blood and 10,896 copies/ml plasma (Figure 1, Tables 2–4). CMV was detected by PCR in one CINS patient and in no healthy control patients (Table 2).

**Table 2:** Frequency of Viral DNA in Septic and Control Patients.

Virus	Septic	Critically-Ill Non-Septic	Healthy Controls
	*No. positive* † */No. tested (%)*		
**CMV** *	86/356 (24.2)	1/89 (1.1)	0/165 (0)
**EBV**	287/539 (53.2)	18/149 (12.1)	6/165 (3.6)
**HSV**	76/538 (14.1)	2/150 (1.3)	0/165 (0)
**HHV-6**	56/539 (10.4)	1/150 (0.7)	7/165 (4.2)
**TTV** ‡	179/231 (77.5)	33/55 (63.6)	98/165 (60.1)
**JC** **	85/238 (35.7)	10/42 (23.8)	
**BK** **	35/237 (14.3)	4/42 (9.5)	
**Any Virus**	432/560 (77.1)	62/161 (38.5)	104/165 (63.0)
**>1 Virus**	239/560 (42.7)	9/161 (5.6)	9/165 (5.5)

† Except where indicated, No. positive reflects the number of patients who tested positive in either whole blood or plasma or both. No. tested represents the total number of patients tested.

*Results are from CMV seropositive patients only.

‡ Tested in plasma only.

**Tested in urine.

**Table 3:** Frequency of Viral DNA in Blood and Plasma Individually.

Virus	Septic		Critically-Ill Non-Septic		Healthy Controls	
	Blood	Plasma	Blood	Plasma	Blood	Plasma
	*No. positive/No. tested (%)*					
**CMV** *	71/345 (20.6)	33/148 (22.3)	1/79 (1.27)	0/29 (0)	0/164 (0)	0/165 (0)
**EBV**	275/522 (52.7)	75/235 (31.9)	15/127 (11.8)	3/55 (5.45)	5/164 (3.1)	1/165 (0.61)
**HSV**	65/521 (12.5)	42/235 (17.9)	2/128 (1.56)	0/54 (0)	0/164 (0)	0/164 (0)
**HHV-6**	35/522 (6.9)	30/235 (12.8)	1/128 (0.78)	0/55 (0)	5/164 (3.1)	6/165 (3.64)
**TTV**		179/231 (77.5)		35/55 (63.6)		98/165 (60.1)
**Any Virus**	312/522 (59.8)	204/235 (86.8)	19/128 (14.8)	36/55 (65.5)	10/164 (6.1)	102/165 (61.8)
**>1 Virus**	106/522 (20.3)	106/522 (20.3)	0/128 (0)	2/55 (3.64)	0/164 (0)	5/165 (3.03)

*Results are from CMV seropositive patients only. No. positive represents the number of patients who tested positive in Blood or in Plasma separately. No. tested represents the total number of patients tested.

**Table 4:** Viral Loads in Blood, Plasma, and Urine.

	Septic					Critically Ill					Healthy Controls				
	Blood														
	CMV	EBV	HSV	HHV-6	TTV	CMV	EBV	HSV	HHV-6	TTV	CMV	EBV	HSV	HHV-6	TTV
**GeoMean**	6409.1	6067.7	6144.2	5863.1	3.65E+05	3091	5631	4000	3000	1.41E+05	N/A	4000	N/A	1.85E+07	63618
**GeoSEM**	1.21	1.06	1.19	1.39	1.2	1	1.29	1	1	1.5	N/A	1	N/A	10.6	1.44
**Median**	2326.5	4000	4000	3000	2.71E+05	3091	4000	4000	3000	2.18E+05	N/A	4000	N/A	2.39E+07	62870
**Max**	554917	1.00E+07	4.29E+07	1.33E+07	5.30E+09	3091	1.44E+05	4000	3000	3.24E+07	N/A	4000	N/A	1.92E+09	2.18E+07
**Min**	2000	4000	4000	3000	2000	3091	4000	4000	3000	2000	N/A	4000	N/A	3000	2000
	**Plasma**														
	**CMV**	**EBV**	**HSV**	**HHV-6**	**TTV**	**CMV**	**EBV**	**HSV**	**HHV-6**	**TTV**	**CMV**	**EBV**	**HSV**	**HHV-6**	**TTV**
**GeoMean**	10896.5	4000	14342.5	4491.3	63946.9	N/A	4000	N/A	N/A	27047	N/A	4000	N/A	1.95E+06	20697
**GeoSEM**	1.41	1	1.25	1.31	1.25	N/A	1	N/A	N/A	1.37	N/A	1	N/A	5.16	1.26
**Median**	3243.4	4000	10640	3000	33504.2	N/A	4000	N/A	N/A	33248	N/A	4000	N/A	1.73E+06	11255
**Max**	435789	4000	1.16E+06	1.41E+06	8.00E+09	N/A	4000	N/A	N/A	1.50E+06	N/A	4000	N/A	1.58E+08	6.50E+06
**Min**	2000	4000	4000	3000	2000	N/A	4000	N/A	N/A	2000	N/A	4000	N/A	3000	2000
	**Urine**														
	**JC**	**BK**				**JC**	**BK**								
**GeoMean**	2.32E+06	62441				9.67E+05	17931								
**GeoSEM**	1.5	1.59				2.89	2.61								
**Median**	2.57E+06	31121				8.84E+05	16706								
**Max**	4.41E+09	7.52E+07				3.56E+08	2.20E+05								
**Min**	4000	4000				9620	4000								

#### EBV

EBV was detected in blood samples from 53.2% of septic patients (Tables 2–4). Fifty-two septic patients (18.9%) had levels ≥10,000 copies/ml whole blood, a level that is considered an indication for reducing immunosuppression in solid-organ transplant recipients at our institution (Figure 1, Table 4). EBV was detected in 12.1% and 3.6% of CINS and healthy control patients respectively.

#### HSV

HSV was detected in 14.1% of septic patients with geomean equaling 6,144 copies/ml whole blood and 14,342 copies/ml plasma (Tables 2–4). HSV was detected in blood of 1.5% of CINS patients. No healthy control patients had HSV viremia.

#### HHV-6

HHV-6 was detected in 10.4% of septic patients (Tables 2–4). One CINS and 6 (3.3%) healthy control patients were positive. HHV-6 levels were generally at or below the lower limit of quantitation of the qPCR assay (3,000 copies/ml blood).

### Cumulative detection rate and levels of TTV

TTV was detected in plasma of 77.5% of septic patients with geomean equaling 64,000 copies/ml (Tables 2–4). TTV was detected in 63.6% and 60.1% of CINS and healthy control patients respectively. Geomean TTV levels were 27,000/ml and 21,000/ml in plasma of CINS and healthy control patients respectively.

### Urine BK and JC detection rates and levels

JC was detected in urine of 35.7% of septic patients with geomean level of 2.3×10^6^ copies/ml (Table 2). JC was detected in 23.8% of CINS patients with geomean level of 9.7×10^5^ copies/ml. BK was detected in urine of 14.3% and 9.5% of septic and CINS patients respectively. BK geomean values were 62,441 copies/ml and 17,931 copies/ml in septic and CINS patients (Table 4).

### Septic patients have multiple viruses with corresponding high viral titers

**Figure 2:**
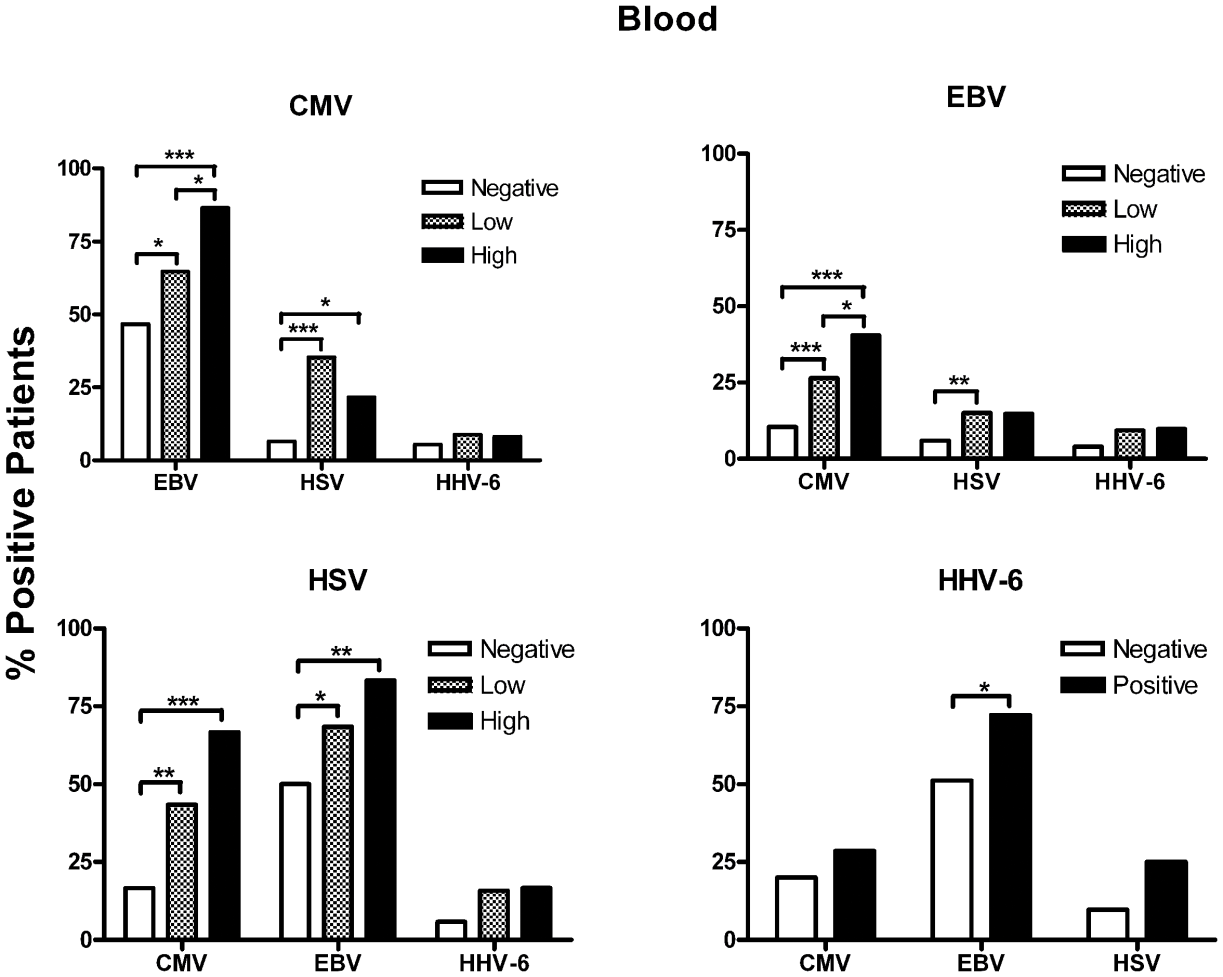
Correlation of viral loads among the individual viruses. Populations were established based upon viral DNA loads; each of these populations was examined for presence or absence of other viruses. The number of patients in each of the groups is defined as the following: *Negative*  =  no detectable virus, *Low*  =  less than lower limit of quantitation (lloq), and *High*  =  greater than lloq. The negative, low, and high values for CMV are N = 274, 34, and 37 septic patients, respectively. Negative, low, and high values for EBV are N = 247, 213, and 61 septic patients, respectively; for HSV comparable values are N = 465, 38, 18, septic patients, respectively). For HHV-6, Negative = no detectable virus (n = 485 patients), Positive = detectable virus (n = 36 patients); *p<0.05, **p<0.01, ***p<0.001. These results show that as the blood viral load of one particular virus increases, there is a corresponding increase in the prevalence of the other herpes family members.

Overall, 42.7% of septic patients had two or more viruses detected during their illness (Table 2). This 42% may underestimate the frequency because not all patients were tested for all viruses. In a subgroup of 209 patients who were tested for all viruses, 54.1% were positive for multiple viruses including 27.8% positive for 2 viruses, 17.2% for 3 viruses, 7.7% for 4 viruses, 3.8% for 5 viruses, and 0.5% for 6 viruses. We also correlated the impact of the load of each of the viruses upon the prevalence of other viruses. In blood samples, the magnitude of the viral load of one herpesvirus often correlated with increased prevalence of other herpesviruses (e.g. it was more common for patients with high CMV loads to have positive EBV tests than it was for patients who had low CMV loads or negative CMV tests), Figure 2. This correlation tended to occur in plasma as well but was not as prominent (Figure S1). This relationship did not hold between the herpes- and polyoma-viruses, i.e. there was no significant relationship between the load of any of the herpesviruses and prevalence of either polyomavirus, and vice versa (data not shown).

### Time course of viral detection

**Figure 3:**
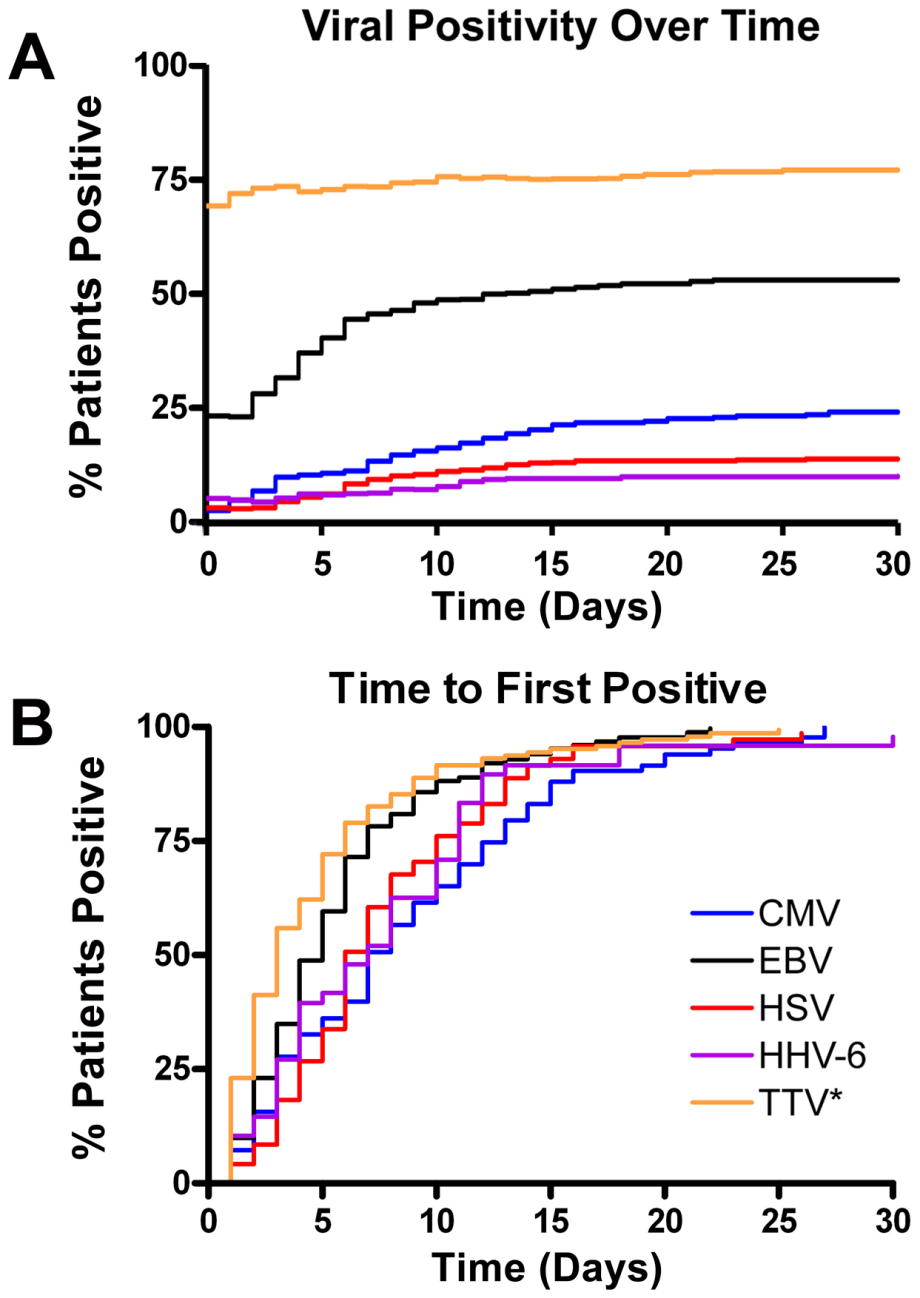
Peak viral detection rate and time course of viral detection. The percentage of patients who tested positive in blood for particular viruses during the course of sepsis (limited to 30 days) is displayed in two formats. Day 0 represents the day that the patient fulfilled sepsis criteria [32]. *Figure 3A* represents all septic patients positive for viral reactivation divided by the total number of septic patients who were tested on or before the same day. *Figure 3B* represents only those septic patients who were negative for the particular viruses and who ultimately became positive during their septic course. The % represents the increase in the number of septic patients who convert from virus negative to virus positive status. *TTV was tested only in plasma (see Methods S1).

During sepsis, virus detection rate increased for all viruses with ICU duration (*Figure 3*). The rapidity at which septic patients who were virus negative at study entry and who converted to virus positive status during their illness differed for various viruses (Figure 3B). The fastest conversion rate occurred for TTV with 50% and 75% detection rates occurring at days 3 and 6 after sepsis onset respectively. Among herpes viruses, the most rapid increase in detection rate (conversion from negative to positive viremia) occurred for EBV with 50% and 75% detection rates of 5 and 7 days respectively. CMV had the slowest rise with 50% and 75% detection rates occurring at days 8 and 13 respectively. The 50% and 75% conversion rates for HSV were 7 and 10 days respectively while those for HHV-6 were 7 and 11 days respectively. Time course for detection of urine BK and JC virus is depicted in *Figure S2*.

### Correlation of viremia with clinical and laboratory parameters

#### Secondary infections

**Figure 4:**
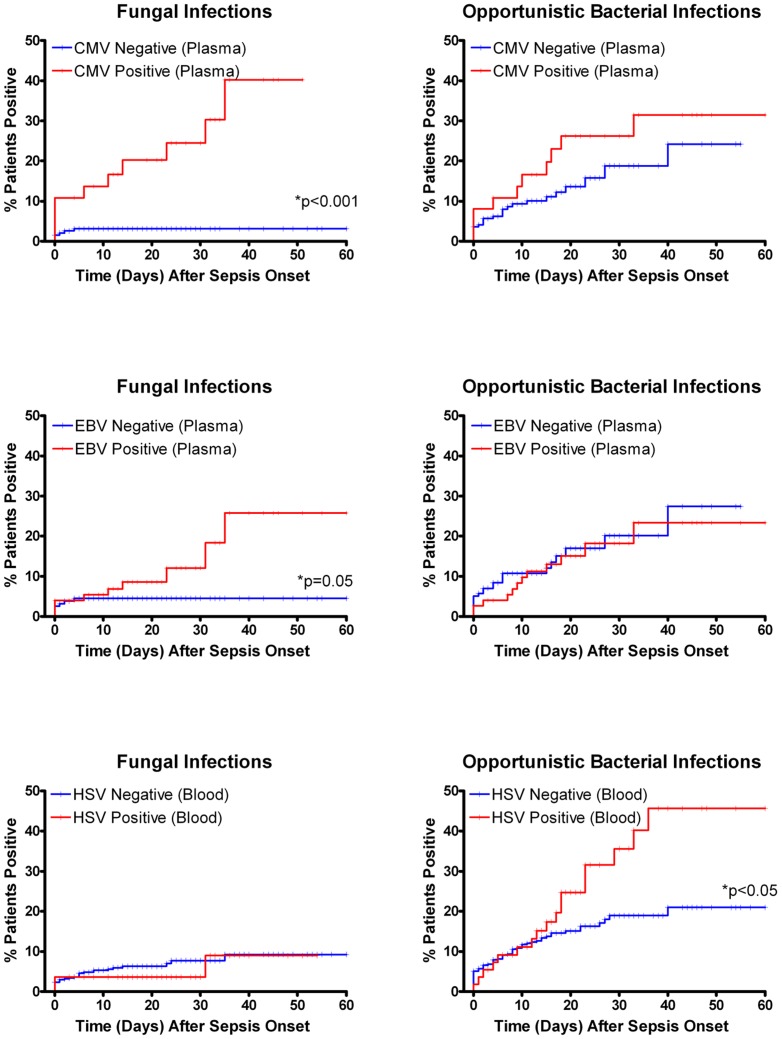
Impact of viral reactivation on fungal and opportunistic bacterial infections. Septic patients with CMV detected in either blood or plasma had increased fungal infections compared to CMV negative patients; only results for plasma are shown and are significant, *p*<0.001. Similarly, patients who had EBV detected in blood had increased fungal infections compared to viral negative patients, *p* = 0.05. Patients who were HSV positive in blood had increased opportunistic bacterial infections due to *Stenotrophomonas*, *Acinetobacter*, or *Enterococcus* compared to viral negative patients, *p*<0.05. Censored subject (vertical hash marks) represent patients who were either discharged from the ICU or who died without events. Analysis was performed using all events but plot was truncated at 60 days for clarity. N = 35 patients with fungal infections, n = 86 patients with *Stenotrophomonas*, *Acinetobacter*, or *Enterococcus* infections.

Impaired immunity in septic patients is frequently manifest by infections with fungal or relatively non-virulent “opportunistic” type bacterial organisms [40], [41]. We prospectively selected *Acinetobacter, Stenotrophomonas, and Enterococcus* as representative of “opportunistic” bacteria in patients with sepsis; these relatively weakly virulent pathogens are common causes of secondary infection in our ICUs [41]. Septic patients who had detectable CMV in either blood or plasma and septic patients who had EBV detectable in plasma had increased risk of fungal infections independent of length-of-stay or duration of sepsis, Figure 4 and Figure S3; (*p*<0.001 for CMV and *p*<0.05 for EBV). For both viruses, the relationship was stronger for detection of virus in plasma than whole blood. These relationships with fungal infection were not present for the other viruses examined. Patients who had detectable HSV in blood had increased risk of developing opportunistic bacterial infections which was independent of length-of-stay, Figure 4, (*p*<0.05). A similar trend was also apparent for detection of HSV in plasma but not for any other virus.

#### ICU duration and severity of illness

**Figure 5:**
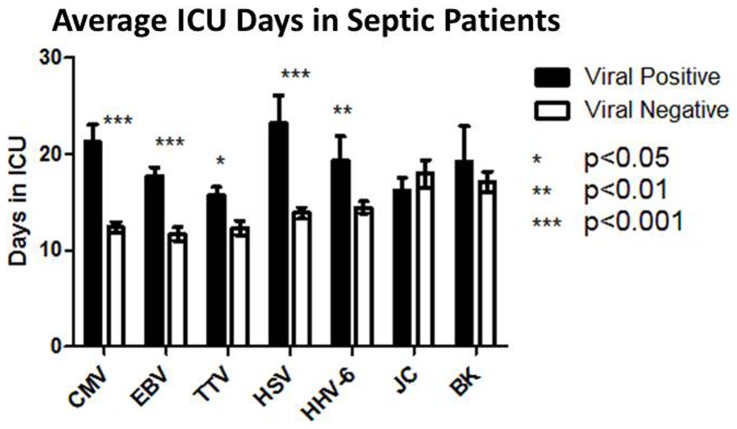
Patients with viral reactivation have increased ICU length of stays. The average number of days spent in the ICU for septic patients with versus without viremia was determined. Septic patients who were positive for CVM, EBV, TTV, HSV, and HHV-6 had longer ICU stays compared to comparable patients who were viral negative. There was no impact of urine JC or BK positivity on ICU length of stay. Values were compared by student's *t* test.

Average ICU length-of-stay was increased in septic viremic versus non-viremic patients, Figure 5. Patient microbiologic data and white blood cell counts are shown in Table 5. For CMV and HSV, the number of ICU days was approximately doubled in patients who were viral positive versus viral negative. No effect of urine BK or JC was observed on length-of-stay. Septic patients with CMV viremia in blood had increased APACHE-II scores compared to CMV negative Table 6, *p*<0.01. Viremia with CMV, EBV, HSV, and HHV-6 was associated with higher SOFA scores, Table 6, *p*<0.01.

**Table 5:** Microbiology and Blood Cell Counts.

		Septic	Critically-ill Non-septic	Healthy Controls
Microbiology	Gram Negative	389		
	Gram Positive	323		
	Fungae	39		
White blood cell count (K/mm^3^)	median	13.1	8.1	6.4
	range (IQR)	10.3–18.9	6.7–9.2	5.5–7.7
Absolute Lymphocytes K/mm^3^	median	0.9	1.1	1.8
	range (IQR)	0.6–1.3	0.7–1.6	1.4–2.3
Absolute Monocytes K/mm^3^	median	0.7	0.6	0.5
	range (IQR)	0.4–1.1	0.4–0.8	0.4–0.6
Absolute Polymorphonuclear K/mm^3^	median	11.8	6	4.1
	range (IQR)	8.5–16.7	5.0–7.4	3.2–5.5

**Table 6:** Correlation of Viral Positivity and Severity of Illness.

	Mean† APACHE II (S.E.M.)			Mean† SOFA (S.E.M.)		
Virus	Virus Positive	Virus Negative	P-value	Virus Positive	Virus Negative	P-value
**CMV** *	18.2 (0.58)	16.3 (0.39)	0.002	9.5 (0.41)	8.3 (0.20)	<0.01
**EBV**	17.2 (0.26)	17.4 (0.33)	0.687	8.9 (0.20)	8.0 (0.20)	<0.01
**HSV**	17.4 (0.49)	17.3 (0.23)	0.346	9.8 (0.42)	8.3 (0.15)	<0.001
**HHV-6**	18.4 (0.77)	17.2 (0.22)	0.145	9.8 (0.42)	8.3 (0.14)	<0.001
**TTV**	16.7 (0.26)	15.9 (0.44)	0.94	8.5 (0.19)	8.2 (0.26)	0.264
**JC**	16.3 (0.49)	16.6 (0.44)	0.783	7.3 (0.35)	7.4 (0.28)	0.963
**BK**	15.4 (0.60)	16.6 (0.37)	0.247	7.2 (0.48)	7.4 (0.24)	0.897

† Mean value represents mean of all patients' average APACHE II or SOFA score for the duration of their ICU stay.

*Represents CMV seropositive patients only.

#### Effect of viral reactivation on mortality in sepsis

**Figure 6:**
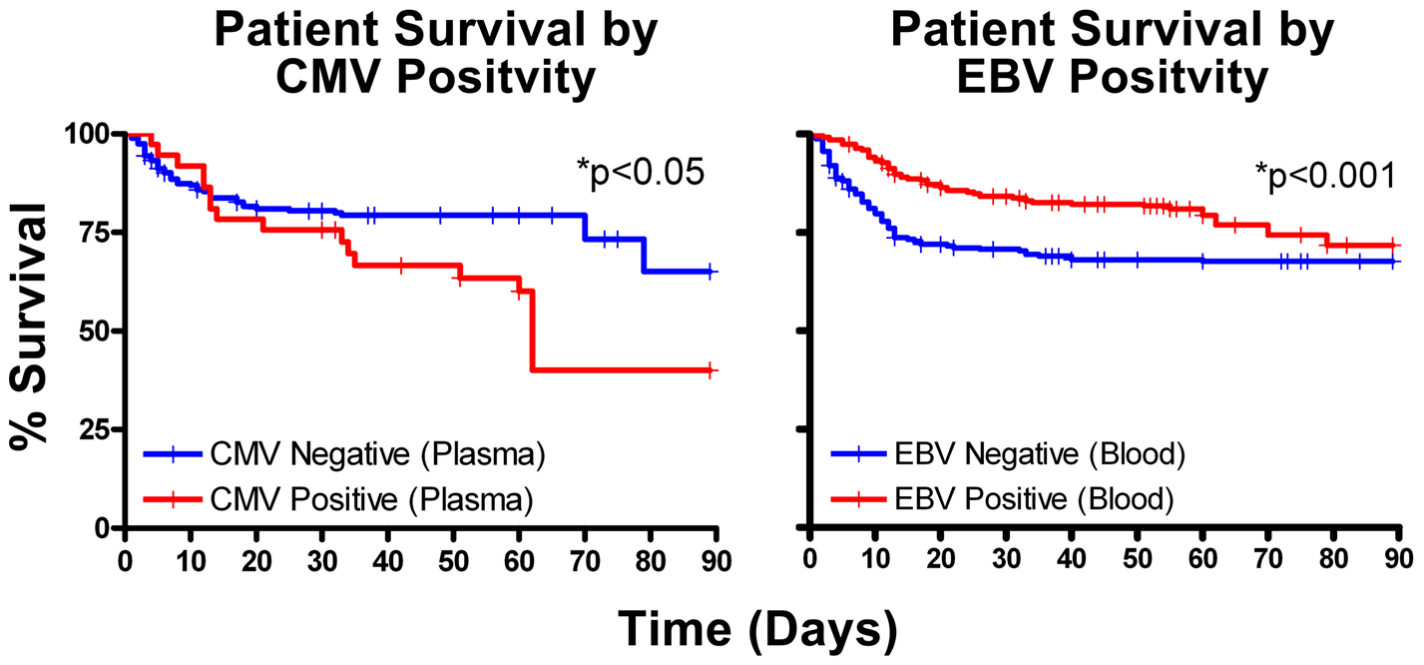
Impact of CMV and EBV on sepsis mortality. Septic patients who were CMV positive in plasma had increased 90 day mortality compared to CMV negative patients, *p*<0.05. Surprisingly, patients who were EBV positive in whole blood (but not plasma) had decreased 90 day mortality compared to EBV negative patients, *p*<0.001. Data analyzed by Kaplan Meier.

**Figure 7:**
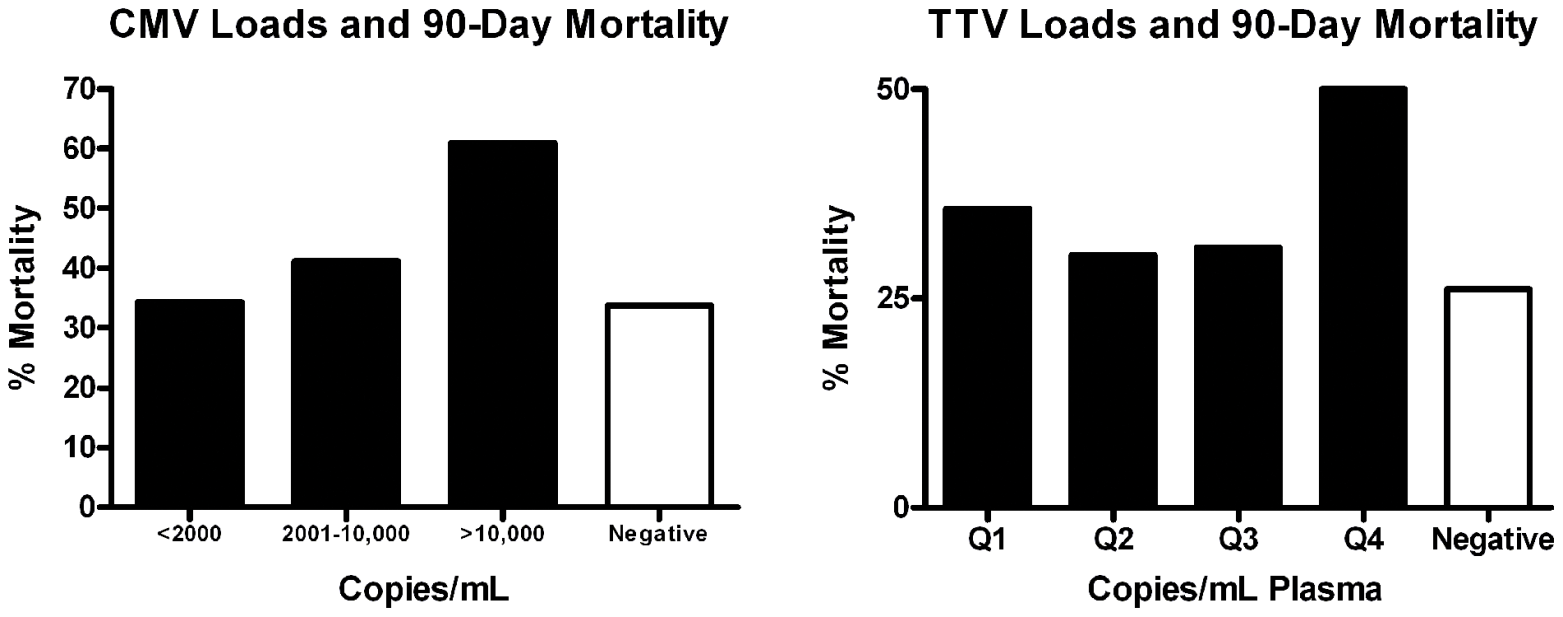
Impact of CMV and TTV viral loads on sepsis mortality. The relationship between CMV and TTV viral load in blood and 90 day mortality is displayed. There was a non-statistically significant increase in mortality due to sepsis with increasing CMV viral levels in blood. (Note that septic patients who were CMV positive in plasma did have increased mortality compared to CMV negative patients - see Figure 6). Compared to septic patients who were TTV negative, patients with the highest quartile viral load for TTV (Q4) had a trend toward increased 90 day mortality (*p* = 0.06).

**Figure 8:**
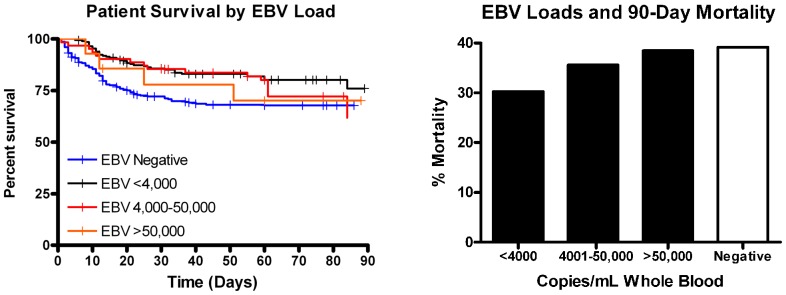
Effect of EBV load on survival. EBV in whole blood (but not plasma) was associated with a decrease in sepsis mortality. This protective effect of EBV DNAemia tended to lessen with increased viral burden although the effect was not statistically significant.

Septic patients with detectable CMV in plasma had increased 90-day mortality compared to CMV negative patients, Figure 6; *p*≤0.05. The increased mortality with CMV had a stepwise increase in mortality with increased viral levels, Figure 7; though this was not statistically significant. Compared to septic patients who were TTV negative, there was a trend for increased mortality in septic patients who had the highest quartile of TTV viral load, Figure 7. Surprisingly, septic patients who were EBV positive in blood (but not plasma) had lower 90-day mortality, Figure 6; *p*<0.05. The protective effect of EBV tended to lessen as viral load increased in whole blood, Figure 8.

## Discussion

A remarkable finding in the present study is the high prevalence of viral DNA in blood of septic patients. Previous studies which investigated viral reactivation in sepsis were generally focused on CMV or, much less commonly, HSV [15], [20], [24]–[28], [42]–[44]. This is the first study to examine the impact of sepsis on multiple families of viruses. Detection of herpes viruses (CMV, EBV, HSV-1, and HHV-6), polyomaviruses (JC and BK), and anellovirus (TTV) occurred with high frequency in sepsis (Tables 2–4). These increased rates of viral detection are particularly striking when compared to results in non-septic patients and healthy-control patients. The fact that 42.7% of septic patients had viremia with multiple viruses as well as the magnitude of viral loads (Figure 1) provides strong evidence that host immunity is impaired in sepsis. Potential mechanisms of immunosuppression in sepsis include T-cell exhaustion, apoptotic depletion of CD4 and CD8 T-cells, myeloid-derived suppressor cells, and increased T-regulatory cells, all of which might contribute to viral reactivation [4], [23], [45], [46]. Importantly, EBV, CMV, and HHV-6 detection rates for septic patients in this study are similar to those reported in stem-cell and organ transplant patients [47]–[50]. For example, a study of solid organ transplant recipients reported detection rates in blood of 56.3% for EBV, 13.7% for HHV-6, 12.2% for BK and 4.9% for JC [47]. Thus, viral detection in septic patients is comparable to that in transplants patients who are pharmacologically immunosuppressed, providing further support that our findings are indicative of clinically-relevant immunosuppression.

The 24.2% incidence of CMV reactivation in sepsis in the present study is similar to other sepsis studies [15], [24], [25], [44], [51]. Although HSV pneumonitis occurs in sepsis [20], [28], [52], the incidence of HSV viremia in sepsis has (to our knowledge) not been previously reported. One study noted a >50% incidence of HHV-6A in critically-ill patients but this study was not confined to septic patients and the high percentage of HHV-6A reactivation seems incongruous with their other study finding of absence of CMV reactivation in their same patients [53]. The incidences of EBV, TTV, JC, and BK have not previously been reported in septic patients and therefore represent an important independent contribution to the literature.

Detection of the various viruses in the present study presumably represents viral reactivation. Almost all adults have been previously infected with HHV-6 and ∼90% of adults have been previously infected with EBV [16], [49]. The seroprevalences for HSV-1 and HSV-2 are 58% and 17% respectively [54] while those for JC and BK are ∼70–80% and 60–70% respectively [17], [19], [55]. Therefore, it is likely that viral detection in the setting of sepsis is not due to primary infection but rather to viral reactivation. The precise mechanisms that lead to reactivation of latent viruses are not completely established, and indeed may differ between the different viruses. Pro-inflammatory cytokines, hypoxia, cell injury, and other stress-related mechanisms can induce viral reactivation and are commonly present in sepsis [56], [57]. Thus, in addition to impaired immune surveillance, the initial hyper-inflammatory septic phase likely provides the stimulus which precipitates viral reactivation. However, the persistence and degree of elevated viral levels suggests that immune function is insufficient to effectively clear the viruses, strongly suggesting immune dysfunction. Most viruses were detected at high levels in plasma as well as blood (Table 2) and this finding is considered indicative of active viral replication [58]. Thus, while stress-induced mechanisms might initiate viral reactivation in sepsis, the predominant driving force for the extent, persistence, and degree of viral reactivation in most septic patients is most likely to be immune dysfunction. The degree and magnitude of viral loads is also consistent with impaired immunity in septic patients (see discussion below for EBV and TTV viral loads and immunosuppression).

EBV blood level is used as a surrogate marker of immunosuppression in transplant patients [49], [59], [60]. Fifty-two septic patients had EBV levels ≥10,000 copies/ml of whole blood, a level that some transplant clinicians consider to represent excessive immunosuppression and therefore advise reduction in anti-rejection medications [60]. Previous studies have also shown correlation between circulating TTV levels and immunocompetence [29]–[31], [61]. Unlike herpes viruses, TTV is not thought to enter latency but rather to actively replicate at low levels and is present in plasma in ∼50% of healthy adults without known pathologic effects [29]–[33]. Previous studies reported that elevated TTV viral loads occur more frequently in hemodialysis patients, diabetics, and HIV-infected patients with low CD4 counts than in healthy individuals or HIV-infected patients with CD4 counts >500/mm^3^ [29]–[33]. Three studies have reported that TTV viremia increases with the degree of immunosuppressive therapy in patients with organ transplantation and suggested that the magnitude of TTV viremia is indicative of the robustness of the immune system [31]–[33]. The high prevalence (76.4%) and viral load of TTV in septic patients likely reflects their immunosuppression.

A critical question which is *not* answered by the present study is whether the increased viral reactivation in sepsis is merely a marker of impaired immunity or contributes to sepsis morbidity/mortality. A subgroup of septic patients had extremely high levels of CMV and/or EBV (Figure 1) which are frequently associated with pathological effects. A current hypothesis is that CMV and HSV reactivation amplify sepsis-induced lung and systemic inflammation thereby contributing to multi-organ failure [15], [61], [62]. Additionally, chronic viral infections lead to T cell exhaustion and impaired immunity [63], and a recent postmortem study of septic patients demonstrated findings highly consistent with T cell exhaustion [23]. Thus, viral reactivation in sepsis could lead to T cell exhaustion which further impairs host immunity leading to additional viral reactivation. Septic patients who had viral reactivation had increased infections with organisms that generally do not infect patients with competent immune systems, e.g. *Candida albicans*, *Stenotrophomonas*, *Acinetobacter, Enterococcus* (Figure 4) [40], [41]. While this commensal fungus and these bacteria are generally considered opportunistic bacteria, they may enter the bloodstream through barrier breakdown. Whether the increased propensity for infections with relatively weakly pathogenic organisms is a result of viral-mediated effects to impair immunity or whether viral reactivation occurs more readily in more profoundly immunosuppressed septic patients is unknown.

A surprising finding is the decreased mortality in septic patients with EBV viremia in blood (but not plasma) compared to EBV-negative patients (Figure 6). A potential explanation for this seemingly paradoxical finding is provided by studies showing that mice with low level gammaherpes-virus-68 infection (a murine virus genetically similar to human EBV) have improved survival and/or decreased microbial burden in bacterial sepsis due to *L. monocytogenes* and *Y. pestis* [64]. In that animal model, EBV infection protected by activating NK cells to produce IFN-γ, an essential factor for viral control. Significantly, EBV in plasma did not display a survival benefit and was associated with increased fungal infections. These findings may signal a fundamental difference between patients with low and high levels of EBV in blood. We speculate that early reactivation of EBV in sepsis identifies patients who mount a more vigorous response to the pathogens. However, persistent EBV at high levels is likely detrimental to the host.

There are several significant implications of the present study. First, the current results highlight a degree of immunosuppression in septic patient that is on par with pharmacologically-induced immunosuppression in organ transplant patients [47]–[50]. Secondly, an intriguing idea is that serial quantitation of circulating viral load for a panel of viruses may be useful as a biomarker of host immunity in sepsis. This concept of tracking changes in viral load is similar to the approach used to guide dosing of immunosuppressive mediations in some organ transplant recipients [47]–[50]. Besides the viruses quantitated in the present study, HHV-7, adenovirus, parvovirus B19, and human bocavirus are other candidates that might provide additional information regarding the status of host immunity [64], [65], [66]. Finally, these results provide a strong rationale for future and ongoing clinical trials of agents that boost host immunity in patients who have entered the immunosuppressive phase of sepsis [11], [12].

A limitation to this study is the inability to make direct comparisons between septic and control groups. The ICU length-of-stay for CINS was considerably shorter than for septic patients because these patients tended to be more clinically stable and were transferred out of the ICU. Consequently, more serial-samples were obtained from septic versus CINS patients, undoubtedly contributing to the increased detection of viral DNA in sepsis. Additionally, severity of illness in septic patients is invariably higher as a consequence of sepsis-induced multi-organ dysfunction. These issues make direct statistical comparisons between septic and control patients invalid. However, 31 CINS patients who became septic during their ICU stay were included and these patients had viral reactivation typical of the septic group at large following sepsis onset. It is possible that viral reactivation may not be related simply to sepsis but could extend to all critically-ill patients with similar severity of illness and length-of-stay. In this regard, EBV reactivation was higher in CINS patients versus healthy controls, *p*<0.003.

## Conclusions

In conclusion, reactivation of latent viruses is extremely common in patients with prolonged sepsis and is consistent with development of immunosuppression. Whether reactivated viruses represent an epiphenomenon or contribute to morbidity and mortality remains unknown and should be addressed because of their potential impact on morbidity and mortality. Serially tracking of viral load for a panel of latent viruses might be useful as indicators of the state of host immunity.

## Supporting Information

Figure S1 **Effect of viral load on prevalence of other viruses.** This Figure corresponds to Figure 2 displaying results for plasma as opposed to blood. Populations were established based upon viral DNA loads; each of these populations was examined for presence or absence of other viruses. Groups are defined as Negative = no detectable virus; Low = less than the median DNA load; High = greater than or equal to median DNA load. Negative, low, and high values for CMV (median = 3,243, n = 115, 16, 17 respectively) and HSV (median = 10,640, n = 193, 21, 21 respectively). For EBV and HHV-6, Negative = no detectable virus (n = 146 and n = 205 respectively), Positive = detectable virus (n = 72 and n = 30 respectively). For TTV, Negative = no detectable virus (n = 52), Q1 = first quartile (<5,881 copies/mL n = 45), Q2 = second quartile (between 5,881 and 33,504 copies/mL, n = 45), Q3 = third quartile (between 33,717 and 299,609 copies/mL, n = 45), and Q4 = fourth quartile (>299,609 copies/mL, n = 44). Although the correlation is not as striking as in blood (Figure 2), there is a correlation between the viruses such that as the level of one virus increases, there tends to be a concomitant increase in the prevalence of other herpes viruses.
(TIF)

Figure S2 **Peak detection rate and time course of detection for BK and JC.** The percentage of patients who tested positive in urine JC or BK virus during the course of sepsis (limited to 30 days) is displayed in two formats. Day 0 represents the day that the patient fulfilled sepsis criteria. *Figure S2A*. represents all septic patients positive for viral reactivation divided by the total number of septic patients who were tested on or before the same day. The plot starts at day 3 because of skewing of display by small patient numbers. *Figure S2B* represents only those septic patients who were negative for the particular viruses and who ultimately became positive during their septic course. The % represents the increase in the number of septic patients who convert from virus negative to virus positive status.
(TIF)

Figure S3 **Percentage of fungal infections in septic patients.** The percentage of hospital-acquired fungal infections at day 60 were quantitated for septic patients with or without CMV and EBV viral reactivation. Note that patients whose blood was positive for CMV or EBV had increased incidence of fungal infections as depicted in the vertical axis. (The data for the relationship between fungal and opportunistic bacterial infections for patients who were positive for CMV or EBV in plasma is shown in Figure. 4. Censored subject (vertical hash marks) represent patients who were either discharged from the ICU or who died without events. Analysis was performed using all events but plot was truncated at 60 days for clarity.
(TIF)

Table S1 **qPCR assays.** Characteristics of virus qPCR assays, including LLOQs (Lower Limits of Quantitation), average CVs and references.
(CSV)

Methods S1 **Supporting materials and methods.** Expands upon inclusion/exclusion criteria, virus qPCR assays and analysis criteria.
(DOCX)
