# Supplementary figures and images for: Reactivation of Multiple Viruses in Patients with Sepsis
Source: PLoS One. 2014 Jun 11;9(6):e98819. doi: 10.1371/journal.pone.0098819 (PMC4053360; doi:10.1371/journal.pone.0098819)

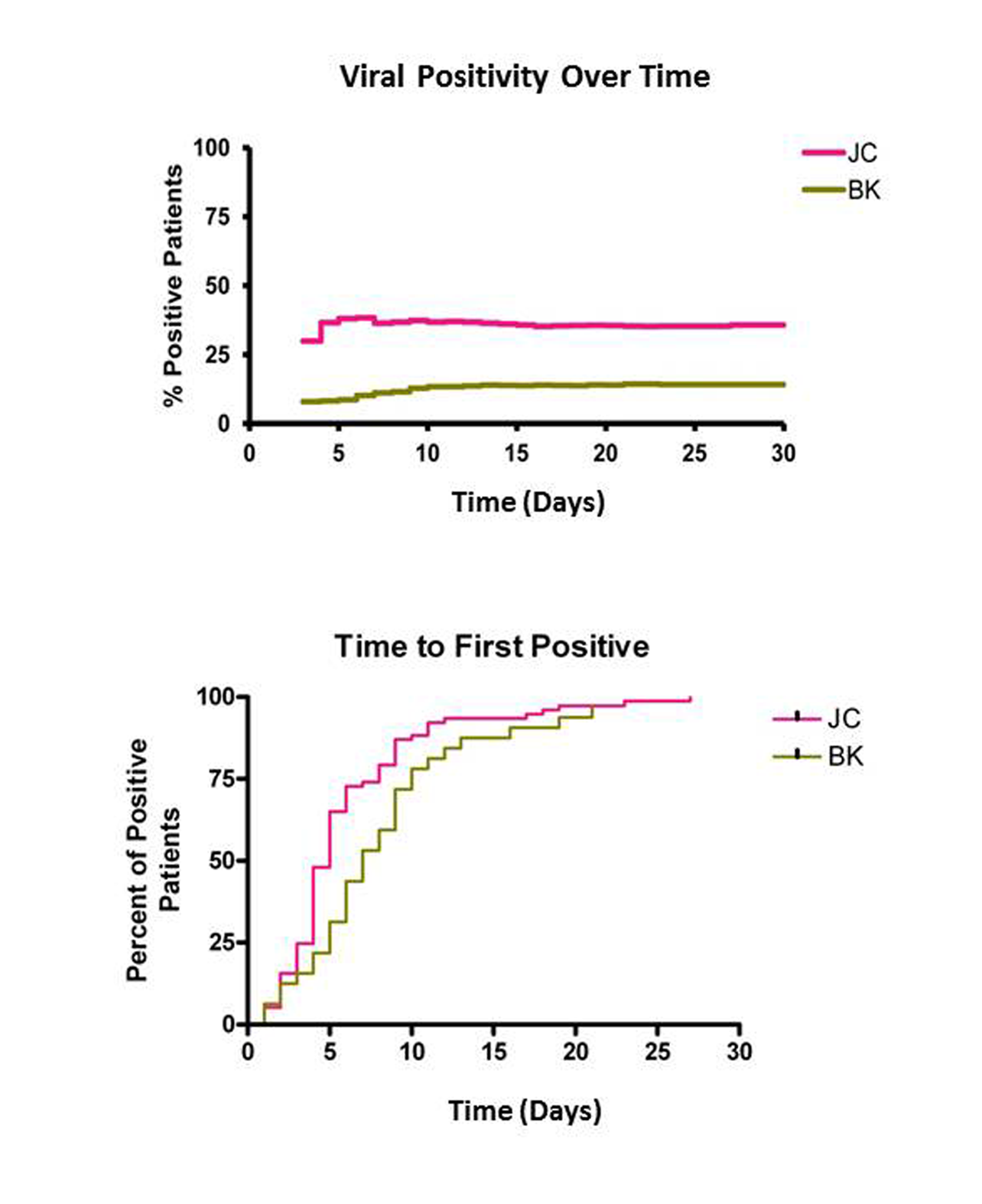

Supplement: Figure S2 — Peak detection rate and time course of detection for BK and JC. The percentage of patients who tested positive in urine JC or BK virus during the course of sepsis (limited to 30 days) is displayed in two formats. Day 0 represents the day that the patient fulfilled sepsis criteria. Figure S2A. represents all septic patients positive for viral reactivation divided by the total number of septic patients who were tested on or before the same day. The plot starts at day 3 because of skewing of display by small patient numbers. Figure S2B represents only those septic patients who were negative for the particular viruses and who ultimately became positive during their septic course. The % represents the increase in the number of septic patients who convert from virus negative to virus positive status. (TIF) [file pone.0098819.s002.tif]
