# Supplementary material for: Reactivation of Multiple Viruses in Patients with Sepsis
Source: PLoS One. 2014 Jun 11;9(6):e98819. doi: 10.1371/journal.pone.0098819 (PMC4053360; doi:10.1371/journal.pone.0098819)
Supplement: Methods S1 — Supporting materials and methods. Expands upon inclusion/exclusion criteria, virus qPCR assays and analysis criteria. (DOCX) [file pone.0098819.s005.docx]

*Supporting Materials and Methods*

*Septic patient inclusion criteria*: Non-immunocompromised patients being treated in surgical or medical ICUs (2009-2013) were identified prospectively. Sepsis was defined using a consensus-panel definition: microbiologically-proven, clinically-proven, or suspected infection and presence of systemic inflammatory response syndrome (SIRS) [32]. Patients were followed through hospital discharge or for 90 days after sepsis onset if discharged from the hospital prior to the 90 day time period. We were unable to obtain 90 day outcome data on 24 of 560 septic patients because they were lost to follow-up. For these 24 patients, their last known date of follow-up was used for all determinations regarding mortality. Finally, 31 patients who were initially classified in the critically-ill non-septic patient category, became septic during their ICU stay. When this occurred, these patients were then re-classified as septic patients.

*Critically-ill non-septic patients (CINS) and healthy control patient inclusion data:* Non-septic, non-immunocompromised patients being treated in surgical/medical ICUs were one comparison group. A second comparison group (Healthy controls) consisted of ambulatory volunteers at the hospital for elective surgery (ASA classification 1-3).

*Exclusion criteria:* Patient exclusion criteria included: HIV-1, organ transplantation, high-dose corticosteroids (≥300mgs/day hydrocortisone) or other immunosuppressive medications, viral hepatitis, and autoimmune diseases.

*Blood and Urine Collection*: Analyses were performed on unused blood remaining after hematologic testing. (Septic and CINS) or blood obtained from otherwise healthy, ambulatory volunteers prior to elective surgery (Healthy control). Within 24-72 hrs of patient ICU admission, blood (collected in EDTA) was retrieved daily Monday through Friday. When quantities allowed, a portion of the whole blood sample was centrifuged for plasma; whole blood and plasma were stored at -80°C. For detection of BK and JC, urine was typically obtained twice/week. Quantitative-PCR was performed on whole blood samples collected on Mondays and Thursdays and on plasma samples collected on Mondays, Wednesdays, and Fridays.

In septic patients, a total of 3582 whole blood samples, 1329 plasma samples, and 588 urine samples underwent quantitative PCR. The mean (+/- SEM) number of samples/patient was 5.9 (+/- 0.1), 2.2 (+/-0.08), and 1.0 (+/-0.06) for blood, plasma, and urine respectively. In critically-ill/non-septic patients, a total of 252 blood, 116 plasma, and 52 urine samples underwent PCR. The mean number of samples/patient was 1.6 (+/-0.05), 0.7 (+/-0.06), and 0.3 (+/-0.07) for blood, plasma, and urine respectively.

*Sample preparation and viral DNA detection:* Samples were extracted on a NucliSens Easy Mag extractor (BioMérieux). 200 uL of whole blood was extracted using the “Specific B” protocol. 200 uL of plasma or urine samples were extracted using the “Generic” protocol. Extracts were eluted into 100 uL.

*Virus PCRs*: Assays were obtained from the Clinical Virology Laboratory at St. Louis Children’s Hospital and are CLIA validated (Except HHV-6 and TTV). CMV [33], EBV [34], HHV-6, and TTV [36] assays were performed on an ABI 7500 Fast system (Applied Biosystems). HSV [35], JC [37,38], and BK [37,38] assays were performed on a LightCycler II (Roche). All herpes virus and anellovirus assays were performed on whole blood and plasma extracts, and polyoma virus assays were performed only urine extracts. Quantitated standards used were acquired from Advanced Biotechnologies Inc. Primers and probes for ABI 7500 based assays were acquired from Applied Biosystems, and primers and probes for LightCycler II based assays were acquired from TIB Molbiol. Results are presented as copy number per mL of original sample. 5uL of extracted sample was used per reaction, making the theoretical lower limit of detection 200 copies/mL. Lower limits of quantitation (lloq) for each assay are described in Table S1 with average interassay Coefficients of Variation of all points on the standard curve. Positive samples at or below lloq were assigned copy numbers equal to lloq. The HSV assay measures both type-1 and type-2, and further distinguishes between the two different viruses. In the present work, the results presented for HSV represent either type-1 or -2. All assays were run in triplicate and the results averaged.

*HHV-6 Assay*: The assay amplifies a 96-bp region of the conserved region of U67. The forward primer (HHV6F) was 5’TTT GCC AGT CAG ACA GTT GTT TC, and the reverse primer (HHV6R) was 5’ TGG CCG CAT TCG TAC AGA (200nM each). Two probes were used, one for Type A (HHV6A) 5’ 6FAM-TAT AAT GCC GCG TTT CCA AAT CTA TTG CCT C-TAMRA (150 nM), and one for Type B (HHV6B) 5’ 6FAM-TAT AAT GCC GCA TTT CCG AAT CTA TTG CCT C-TAMRA (60 nM). Standard ABI 7500 cycling protocol was used (2’ @ 50° C, 10’ @ 95° C, 45 cycles of 15” @ 95° C and 1’ @ 60° C). Subsequent analysis of positives allowed for distinguishing between Type A and B, though results here represent either A or B.

*TTV viral testing*: Preliminary studies testing TTV standards in whole blood extracts demonstrated that the assay was reproducible but the maximum value was lower than expected, consistent with an interference by blood elements. Despite this effect, the assay was reproducible and whole blood TTV values tended to track the TTV values in plasma. Therefore, we believe that the TTV values from whole blood served as useful trends for TTV in patient blood samples. However, the values determined in whole blood likely underestimate the extent of the viral load. Therefore, we elected to only report TTV viral load in plasma although the values for TTV in whole blood are provided in Table 4.

*CMV serologic testing*: Serologic testing was performed on the first blood sample available after ICU admission. Serologic testing for IgG antibody to CMV was determined by ELISA technique using kit purchased from Diagnostic Automation/Cortez Diagnoistics, Inc., Calabasas, CA. 10 microliter serum samples were diluted with 200 microliter sample diluent (supplied in kit). 100 microliters of diluted samples were pipetted into wells of pre-coated plate (supplied in kit). ELISA procedure followed according to kit instructions.

*Diagnosis of secondary infections*: Given their low virulence and the fact that these organisms rarely infect individuals with normal immune systems, *Acinetobacter*, *Stenotrophomonas*, and *Enterococcus* were considered to be opportunistic pathogens [39]. The CDC criteria for hospital acquired infections ([www.cdc.gov/hai/](http://www.cdc.gov/hai/)) was used for diagnosis of infections. Isolation of these bacteria from blood, respiratory secretions obtained by bronchoalveolar lavage or deep tracheal suctioning from patients with a diagnosis of new onset ventilator associated pneumonia, newly placed abdominal drains in areas of suspected abscess, cultures of pancreatic tissue or peritoneal fluid, urine from patients with confirmed or suspected new onset urinary tract infection (for *Enterococcus* only), or surgical tissue biopsy, the diagnosis were considered positive for active infection. We did not make a diagnosis of opportunistic infection if the organisms were obtained from respiratory secretions but the patient did not have new onset ventilator associated pneumonia. Ventilator associated pneumonia was diagnosed as per Skrupky et al *Critical Care Med. 40:281-4; 2012*. In the case of *Enterococcus*, we did not make a diagnosis of opportunistic infection in patients who had enterococcus isolated from stool. We also did not make a diagnosis of opportunistic infection in patients with *Enterococcus* in urine in the absence of evidence of new onset urinary tract infection. A diagnosis of fungal infections was made in patients whose blood, tissue, or newly placed abdominal drain cultures were positive for fungal organisms. Patients who had fungal organisms cultured from respiratory secretions or urine were not considered to be infected but rather colonized. The grading of whether patients had opportunistic or fungal infections was done by an investigator who was blinded to the viral status of the patient. Some septic patients were transferred from outside hospitals to Barnes Jewish Hospital. The records from the outside hospital were reviewed and the duration of sepsis was determined based upon review of these records including the outside physician’s diagnosis of sepsis. In those cases in which the duration of sepsis was not clearly indicated in the outside hospital records, the patient’s onset of sepsis was classified as beginning on the day of admission to the Barnes Jewish Hospital ICU.

*Statistical analysis*: Statistical analyses were performed in the Dept. of Medicine’s Biostatistical Consulting Center using SAS Statistical Software Version 9.3. Kaplan-Meier survival analyses were used to test differences in mortality, ICU length of stay, and secondary infection rates. Chi-square and t-tests were used for categorical and continuous variables.

*Human Studies Human Studies:* The study was approved by the Washington University Human Research Protection Office. Patient consent was obtained for venipuncture and chart review from pre-operative elective surgery patients. Oral consent was documented by having the patient sign the study consent form which was then placed in the patient chart with an additional copy kept with the research nurse coordinator. For septic and critically-ill non-septic patients, a waiver of consent was granted for obtaining excess clinical “waste” laboratory blood (that was slated to be discarded) and for review of their relevant hospital records because these procedures were considered to represent minimum risk to the patients.
